# How to manage food allergy in restaurants, cafeterias and fast food outlets?

**DOI:** 10.1186/2045-7022-1-S1-S21

**Published:** 2011-08-12

**Authors:** M Hazel Gowland

**Affiliations:** 1 Allergy Action, St Albans, UK

## 

Increasing numbers of consumers with food allergies encounter challenges when eating food from restaurants, cafeterias and fast food outlets, and also when choosing food sold without factory packaging. Trends have changed in recent decades. We prepare less food at home from ingredients, so that younger generations have fewer preparation skills and limited food knowledge. At the same time, we travel more widely, and enjoy experiencing dishes which originate around the world served by our local food businesses.

Those diners who need to avoid particular foods to protect their health face a range of practical difficulties, starting with the need to find out exactly what dishes contain as ingredients, and then whether the food business has managed to prepare a particular item or dish without contamination from the allergen to be avoided, sometimes in trace quantities. Evidence from fatal reactions to foods and also reported by patient group members indicates that the most important factor in effective avoidance is access to the CORRECT information about what food contains. Most fatal reactions involve consumption of food containing the culprit allergen. In some cases, the allergic person was not actively avoiding the particular allergen, perhaps through lack of accurate diagnosis or avoidance advice, but even those with accurate diagnoses and well-honed avoidance skills have been caught out.

Typical barriers include: not retaining the correct information, poor information-sharing between preparation and service staff, inconsistent recipes, menu words from other languages / cultures which are not understood by staff / customers, cross-contamination in preparation or service, including shared utensils on self-service buffets, customers being nervous about asking for information in front of their friends or colleagues, and not wanting to make a fuss.

This presentation will present case files, practical risk assessment and some examples of effective solutions to protect food allergic consumers eating catered food.

